# Correction: Determinants of change in long-acting or permanent contraceptives use in Ethiopia; A multivariate decomposition analysis of data from the Ethiopian demographic and health survey

**DOI:** 10.1371/journal.pone.0229349

**Published:** 2020-02-12

**Authors:** Gedefaw Abeje Fekadu, Akinyinka O. Omigbodun, Olumuyiwa A. Roberts, Alemayehu Worku Yalew

There are errors in Table 1. The values under headings Percent of women in 2005 and 2016 are misaligned and do not correspond to the correct group under the heading Characteristics of women. Please see the correct Table 1 here.

**Table 1:** Percentage distribution of selected characteristics of married or in-union reproductive-age women in Ethiopia from the 2005 and 2016 Ethiopian Demographic and Health Surveys.

Characteristics of women	Percent of women in	
	2005	2016
Age*		
15–24	24.0	21.1
25–34	38.9	42.8
35–49	37.0	36.1
Place of residence***		
Urban	11.4	16.5
Rural	88.6	83.5
Religion		
Orthodox	46.3	41.6
Muslim	31.8	33.6
Other	21.9	24.8
Education status***		
No formal education	78.1	62.1
Primary	15.2	27.6
Secondary or higher	6.7	10.3
Working status**		
Working	74.2	68.5
Not working	25.8	31.5
Age at first cohabitation***		
Less than 20	84.6	79.1
20 or more	15.4	20.9
Ideal number of children**		
No child	11.8	9.0
1–5	48.9	54.3
6 or more	39.3	36.7
Concordance on number of children***		
Both wants the same	32.7	39.2
Husband wants more	17.1	25.6
Husband wants fewer	4.7	7.2
Do not know	4.5	28.0
Visit by health worker		
No	91.8	70.5
Yes	8.2	29.5

Note: Chi-square:

*Significant at 0.05,

**Significant at 0.01,

***Significant at<0.001

There are errors in Table 2. The values under headings Percent of women using LAPMs in 2005 (n = 7,918) and 2016 (n = 9,127) and the heading Percent change are misaligned and do not correspond to the correct group under the heading Characteristics of women. Please see the correct Table 2 here.

**Table 2:** Long-acting and permanent contraceptive methods use among married or in union reproductive-age women in Ethiopia and percent change by selected characteristics, 2005 and 2016 Ethiopian Demographic and Health Survey.

Characteristics of women	Percent of women using LAPMs in		Percent change
	2005 (n = 7,918)	2016 (n = 9,127)	
Age			
15–24	0.3	10.6	10.3
25–34	0.3	13.8	13.5
35–49	1.2	9.6	8.4
Place of residence			
Urban	4.1	17.7	13.6
Rural	0.2	0.4	0.2
Religion			
Orthodox	1.0	15.3	14.3
Muslim	0.3	5.4	5.1
Other	0.6	13.8	13.2
Education status			
No education	0.3	10.9	10.6
Primary	0.9	10.9	10.0
Secondary or higher	3.7	17.2	13.5
Working status			
Working	0.6	10.4	9.8
Not working	0.7	14.2	15.5
Age at first cohabitation			
Less than 20	0.6	11.5	10.9
20 or more	1.1	11.8	10.7
Ideal number of children			
No child	0.4	6.3	5.9
1–5	0.9	15.2	14.3
6 or more	0.4	9.0	8.6
Concordance on number of children			
The same	0.7	14.3	13.6
The husband wants more	0.5	10.1	9.6
The husband wants less	1.3	10.7	9.4
Visited by health worker in the last 12 months			
No	0.5	10.9	10.4
Yes	1.7	13.3	11.6

There are errors in Table 3. The values under headings Percent difference in LAPMs use due to Characteristics (E) and (C) and Total are misaligned and do not correspond to the correct group under the heading Characteristics of women. Please see the correct Table 3 here.

**Table 3:** Decomposition of the change in long-acting and permanent contraceptive methods use among married or in union reproductive-age women in Ethiopia by selected characteristics, 2005 to 2016.

Characteristics of women	Percent difference in LAPMs use due to		
	Characteristics (E)	Coefficients (C)	Total
Age (in years) at the time of the survey			
15–24	Ref.	Ref.	Ref.
25–34	-0.7	6.8	6.1
35–24	0.4	-10.0*	-9.6
Overall	4.4	-43.9*	-39.5
Age (in years) at first cohabitation			
<20	Ref.	Ref.	Ref.
20 or more	0.5	-0.9	-0.4
Overall	0.3	-1.4	-1.1
Religion			
Orthodox	Ref.	Ref.	Ref.
Muslim	2.6	-9.4*	-6.8
Other	0.3	7.3*	7.6
Overall	4.3	13.1	17.4
Education status			
No education	Ref.	Ref.	Ref.
Primary	1.5	-8.4**	-6.9
Secondary	-0.1	-4.6***	4.7
Above secondary	-1.6	-1.5***	3.1
Overall	-6.1	-10.7***	-16.8
Working status			
Working	Ref.	Ref.	Ref.
Not working	-0.7	4.2	3.5
Overall	-3.9	5.2	1.3
The ideal number of children			
No child	Ref.	Ref.	Ref.
1–5	-2.4	10.8	8.4
6 or more	0.2	5.8	6.0
Overall		-0.5	0.2
Concordance on the number of children			
Both want the same	Ref.	Ref.	Ref.
Husband want more	1.4	-1.3	0.1
Husband want fewer	0.5	-2.2	-1.7
Overall	7.5	-15.8	-8.3
The desire for more children			
Want within two years	Ref.	Ref.	Ref.
Want after two years	-0.3	-2.7	3.0
Other	-1.2	-3.7	4.9
Overall	-3.7	-7.1	-10.8
Visited by health worker			
No	Ref.	Ref.	Ref.
Yes	-0.5	-2.7	3.2
Overall	-5.4	-2.7*	-8.1
Total	7.5	92.5	100.0

Note:

*Significant at 0.05,

**Significant at 0.01,

***Significant at<0.001, ref: reference, LAPM: long acting and permanent methods
