# Supplementary material for: Determinants of change in long-acting or permanent contraceptives use in Ethiopia; A multivariate decomposition analysis of data from the Ethiopian demographic and health survey
Source: PLoS One. 2020 Jan 14;15(1):e0227218. doi: 10.1371/journal.pone.0227218 (PMC6959602; doi:10.1371/journal.pone.0227218)
Supplement: Attachment — Submitted filename: Reviewer_comment.docx [file pone.0227218.s001.docx]

**Reviewer’s report**

**Title:** Determinants of change in long-acting and permanent contraceptive methods use in Ethiopia; a multivariate decomposition analysis of data from the Ethiopian demographic and health survey

**Version: Date**: 17^th^ September 2019

**Reviewer:** Samuel Bosomprah

**Reviewer’s report**

The paper sought to explain the change in LAPMs use over time. I believe that this is an important topic especially in developing countries where there is concern for population growth and its negative consequences on available resources and economic growth. The choice of decomposition analysis technique is appropriate to answer to the research question “why has LAMPs use change over time?”. The paper can be improved if the authors addressed the following concerns.

**Major Compulsory Revisions:** Yes

**Introduction**

Delete the first para of the introduction – it’s just a background. Begin the introduction with a clear declarative statement of the problem. The rest of the sentence in the paragraph should be an elaboration of the problem using global and sub-Saharan statistics.

Para 1 (problem statement):

Rewrite lines 57 to 71 into para 1 as follows:

“Unintended pregnancy is a major global health problem among sexually active women. <describe the global and sub-Saharan LAPMs uptake or use using statistic>. The long acting and permanent contraceptive methods (LAPMs) such as intrauterine devices or IUD, and implants are known to be efficient and cost-effective interventions for reducing unintended pregnancies. Several studies have identified <knowledge about LAPMs, age, education, place of residence, list the rest factors) as factors independently associated with LAPMs use (ref).

Para 2 (justification/significance/rationale/importance): This para should describe why it is important to carry out this study. In otherwise, argue for the study. You may beef up lines 72 to 79 to reflect this thinking.

Para 3 (aims/research questions/objectives/hypotheses (if any): Conclude the introduction with the aims or objectives of the study. And indicate the potential impact of the findings from the study. Lines 80 to 88 could remain as is.

**Methods**

Data analysis: A number of decomposition analysis techniques exist. The authors should acknowledge same and describe which of these is implemented in the mvdcmp command. Recheck the equation in line 151.

**Results**

1. The tables are fragmented. The authors should collapse Tables 1 to 4 into one or two (because independent variables are many) tables. See Table 1 of the paper by Bosomprah et al 2014 (ref 23 in the current paper). In this table, the population structure was presented in the same table as the prevalence. Your last column should be maintained as the Percentage change. Include the “total” row for all tables.
2. The potential factors/drivers are too many. Authors should consider including those found in the literature to be predictors of LAPMs use or uptake. For example, I am not sure whether “sex of head of household” is a predictor of LAPMs use. Also, how is working status different from occupation? Are they not measuring the same thing? Some variables can be combined into a derived variable that makes sense. For example, can we combine: Visited by health worker in the last 12 months; Visited health facility in the last 12 months etc. I can’t see an important predictor like “knowledge about LAPMs”
3. Delete line 174 - this style is thesis report. Rather comment and put the table reference in bracket.
4. Authors should be consistent in the number of decimal places. Authors should keep the percentages in 1 decimal place in the table and the main text.
5. The comments on Table 5 (Decomposition analysis) are confusing. For example, Where are the E and C in Table 5 -as I indicated earlier Authors should include the “Total” column which should have the total E and C. Then the “coeff” are the part of the total E or C which are due to the respective predictors, also translated into percentages.

**Discussion**

The authors should discuss the strength and limitations of the study. They should also discuss the impact of the findings as well as prescription of future work.

**Discretionary Revisions:** Yes

**Level of interest:** An article of importance in its field

**Quality of written English:** Authors should proofread and correct for grammatical errors

**Statistical review:** No, I am a statistician and have reviewed the statistical methods used.

**Declaration of competing interests:** I declare that I have no competing interest
