# Supplementary material for: Determinants of change in long-acting or permanent contraceptives use in Ethiopia; A multivariate decomposition analysis of data from the Ethiopian demographic and health survey
Source: PLoS One. 2020 Jan 14;15(1):e0227218. doi: 10.1371/journal.pone.0227218 (PMC6959602; doi:10.1371/journal.pone.0227218)
Supplement: Attachment — Submitted filename: Response to Reviewers.docx [file pone.0227218.s002.docx]

**Point by point response**

| **S.no** | **Reviewers’/Editor’s comments** | **Authors response** |
| --- | --- | --- |
|  | **Editor’s comments** |  |
| 1 | Please ensure that your manuscript meets PLOS ONE’s style requirements, including those for file naming …. | Thank you for the comment. The manuscript meets the PLOS ONE’s requirements. |
| 2 | We suggest you thoroughly copyedit your manuscript for language usage, spelling, and grammar. …. | Thank you for the suggestion. We thoroughly copyedited the manuscript using online soft wares. |
| 3 | We noticed you not appear to account for multiple comparisons during your statistical analysis. Please provide justification for not doing so or add these to the manuscript. | Thank you for the suggestion. We did not do multiple comparisons because adjusting p value for multiple comparisons may penalize an association being found in large study. Our study based on nationally representative data with big sample size. Instead of that we reported the p values. For some of the variables, the p-value is smaller. Further adjustment cannot yield values smaller than this one. |
| 4 | Please also discuss the possible limitations of your study. | Thank you for the comment. Limitations of the study are included at the end of the discussion section of the revised manuscript. |
| Reviewer 1/Samuel Bosomprah’s comments | | |
| 1 | Delete the first para of the introduction – it’s just a background. Begin the introduction with a clear declarative statement of the problem. The rest of the sentence in the paragraph should be an elaboration of the problem using global and sub-Saharan statistics. | Thank you for the constructive comment. The first paragraph is deleted. |
| 2 | Para 1 (problem statement):  Rewrite lines 57 to 71 into para 1 as follows:  “Unintended pregnancy is a major global health problem among sexually active women. <describe the global and sub-Saharan LAPMs uptake or use using statistic>. The long acting and permanent contraceptive methods (LAPMs) such as intrauterine devices or IUD, and implants are known to be efficient and cost-effective interventions for reducing unintended pregnancies. Several studies have identified <knowledge about LAPMs, age, education, place of residence, list the rest factors) as factors independently associated with LAPMs use (ref). | Thank you for the constructive comment. I revised the paragraph based on your comments. References are also added. |
| 3 | Para 2 (justification/significance/rationale/importance): This para should describe why it is important to carry out this study. In otherwise, argue for the study. You may beef up lines 72 to 79 to reflect this thinking. | Thank you for the constructive comment. It is revised as per your recommendation. |
| 4 | Para 3 (aims/research questions/objectives/hypotheses (if any): Conclude the introduction with the aims or objectives of the study. And indicate the potential impact of the findings from the study. Lines 80 to 88 could remain as is. | Thank you for the constructive comment. It is revised as per your recommendation. |
| 5 | **Methods**  Data analysis: A number of decomposition analysis techniques exist. The authors should acknowledge same and describe which of these is implemented in the mvdcmp command. Recheck the equation in line 151. | Thank you for the comment. We used multivariate decomposition analysis technique, specifically the standard or two-component decomposition technique. We checked the equation; it represents the very beginning of the regression equation. |
| 6 | The tables are fragmented. The authors should collapse Tables 1 to 4 into one or two (because independent variables are many) tables. See Table 1 of the paper by Bosomprah et al 2014 (ref 23 in the current paper). In this table, the population structure was presented in the same table as the prevalence. Your last column should be maintained as the Percentage change. Include the “total” row for all tables. | Thank you for the constructive comments. Tables 1 to 4 are collapsed to two tables. Some less important independent variables are deleted.  Total row is included for table 3. |
| 7 | The potential factors/drivers are too many. Authors should consider including those found in the literature to be predictors of LAPMs use or uptake. For example, I am not sure whether “sex of head of household” is a predictor of LAPMs use. Also, how is working status different from occupation? Are they not measuring the same thing? Some variables can be combined into a derived variable that makes sense. For example, can we combine: Visited by health worker in the last 12 months; Visited health facility in the last 12 months etc. I can’t see an important predictor like “knowledge about LAPMs” | Thank you for the comment. Less important variables were deleted from table 3. Regarding knowledge about LAPMs, the DHS measures knowledge about modern contraceptives, not specifically knowledge about LAPMs. That is why we did not include that variable in the analysis. According to the definition used in DHS, contraceptive knowledge was universal in Ethiopia. |
| 8 | Delete line 174 - this style is thesis report. Rather comment and put the table reference in bracket. | Thank you for the comment. It is deleted now. |
| 9 | Authors should be consistent in the number of decimal places. Authors should keep the percentages in 1 decimal place in the table and the main text. | Thank you for the comment. All percentages are arranged to one decimal place now. |
| 10 | The comments on Table 5 (Decomposition analysis) are confusing. For example, Where are the E and C in Table 5 - as I indicated earlier Authors should include the “Total” column which should have the total E and C. Then the “coeff” are the part of the total E or C which are due to the respective predictors, also translated into percentages. | Thank you for the comment. In the revised table, we added the percentages only leaving the coefficients. The total E and C are presented in the last row of the table. A total row is added for each variable. |
| 11 | The authors should discuss the strength and limitations of the study. They should also discuss the impact of the findings as well as prescription of future work. | Thank you for the comment now we have included strength and limitations of the study. |
| 12 | Authors should proofread and correct for grammatical errors | Thank you for the comment. We now proofread and corrected the grammatical errors. |
| Reviewer 2’s comments | | |
| Introduction | | |
| 1 | The authors need to consider having the manuscript edited before submission. There are several errors related to grammar and punctuation that would be caught by an editor and will also improve readability. | Thank you for the comment. We thoroughly edited the manuscript for grammatical, typo and punctuation errors. We consulted other colleagues to correct such errors. We also used free online tools for the correction. |
| 2 | At some point in the introduction, the authors should provide a definition for Long acting and permanent contraceptive methods (LAPMs). They provide examples, which do help to make a distinction, but a definition will be more appropriate, I think. A definition will also help the authors make a case for why LAPMs are superior to other forms of modern contraceptives. I assume the authors are working on the idea that LAPMs are better for efforts to lower fertility rates in Ethiopia. | Thank you for the comment. we added a sentence defining LAPMs in the introduction section of the revised manuscript. |
| 3 | The authors should decide on the preferred acronym and then stick with it. Throughout the paper, they go between LAPM and LACPM. | Thank you for the comment, we used the acronym “LAPM/LAPMs” throughout the manuscript. |
| Methods | | |
| 4 | The authors should consider providing a definition of women who are of reproductive age (perhaps base it on the Ethiopian government's policy on national fertility). It is important to provide such a definition as that appears to be the primary inclusion criteria for data. For example, the women's survey component of DHS data typically includes women from 15-49 years as is the case with Ethiopia. That age range falls into the reproductive age range. The authors need to confirm if this matches their notions of reproductive age ranges. | Thank you for the comment. The definition for women of reproductive age in Ethiopia is the same as the definition used in DHS. That is, women of reproductive age includes women aged 15 – 49. We included this in the revised manuscript. |
| 5 | The authors chose to limit their data to married women or women cohabiting with a partner, excluding single, divorced and widowed women. I think an explanation is in order here. Contraceptive use is not something that is limited to married women and women in long-term partnerships. I can understand excluding women who are pregnant at the time of the survey, but why exclude the others? Are not the sexual health and reproductive needs of women not living with a permanent partner, pertinent to understanding the use of LAPMs? | Thank you for the comment. It is true that contraceptive use is not something that is limited to married women only. Contraceptive use analysis can be done for married women only, for unmarried sexually active women only or for both. We restricted the analysis to married women for the following reasons.   1. Contraceptive use information among unmarried women is less reliable due to social desirability bias. 2. Unmarried women in Ethiopia, although sexually active, do not report that they are sexually active as sexual activity for non-married is not socially acceptable. 3. Unmarried women are less likely to use LAPMs. In the Ethiopian context, almost all LAPMs use is among married women. |
| 6 | The last paragraph of the data analysis section could be shortened a bit. For example, state from the outset that STATA was the preferred analytical software, and then note that 2005 data was appended to 2016 data to create the analysis data. | Thank you for the comment. We shorted the last paragraph although some readers may need the details of the analysis. |
| 7 | Don't think there is a need to go into detail about how the data was analysis data set was created and what commands were relevant for that process. | Thank you for the comment. It is indicated in the revised manuscript from line 149 to 157. |
| Results | | |
| 8 | I would recommend for this manuscript the authors place the tables at the end of the document rather than incorporate them within the text. It will help improve readability I believe at this stage of the review (minor issue). | Thank you for the comment. We prepared the manuscript based on the journal’s submission guidelines. The guideline recommend authors to place each table in the manuscript directly after the paragraph in which it is first cited. Therefore, we included the tables within the text based on the journal’s recommendation. |
| 9 | The authors use too many decimal places in their tables, particularly in what is Table 05. I can understand having several decimal places for regression coefficients, particularly if the value is small (3 decimal places should be adequate), but for percent values, these can be limited to one or two decimal places. | Thank you for the comment. The decimal places are now corrected and limited to one decimal place. |
| 10 | Consider merging Table 01 and Table 02 and within the table distinguish between the three broad categories of explanatory variables (independent variables), rather than have a separate table for demographic characteristics, a separate table for fertility-related characteristics and a separate table for family planning exposure through media. It will be a long table, but it will improve the way in which readers navigate the paper. | Thank you for the comment. Tables one and two are merged now. Some less important variables are also deleted from the table. |
| 11 | Similarly, the authors should consider merging Table 03 and Table 04. As suggested above, within this table the authors would distinguish between the three broad categories of independent variables. (If the authors are feeling adventurous, it may even be possible to merge Table 01, 02, 03, and 04 into one table that is both wide and long. This table would have one major column that would be characteristics by year and then there would be two sub-columns for 2005 data and 2016 data. Similarly, there would be a larger column for Percent LAPM use per year and then sub column heading for changes in LAPM use). Overall, the authors need to be careful about the column headings and subheadings they are using for their tables. For example, in Table 03, column headings are simply 2005 N=7918 and 2016 N=9127, when the context suggests that the authors mean, percent of LAPM use in those particular years. Another example, in Table 05 which shows the results from the decomposition analysis, the first column is labeled as ‘LAPM contraceptive method use'. I imagine the authors simply mean variable for this particular column heading. | Thank you for the constructive comments. Tables 3 & 4 are merged now. Column headings and subheadings are revised. We did not find merging tables one to 4 to one table interesting. The information will be bulky and difficult to comprehend. |
| 12 | Lastly, does STATA not provide the totals for each variable when looking at differences in characteristics and differences in coefficients/effects? For example, there should be a total age (in years) when reporting differences in characteristics and differences in effects. There should also be an overall total for each column in Table 05. That is there should be totals reported for all differences in characteristics and differences in effects, this would correspond to what was reported in paragraph 2 of the sub-section on decomposition analysis in the results section. | Thank you for your constructive comment. We revised table 5(now table 3) and added row totals and the overall total in the last row. |
| Discussion | | |
| 13 | My only issue with the discussion section at present is that the authors should also consider the practical significance of their findings. Statistical significance is certainly important and worth noting and explaining. However, LAPMs in the context of a country like Ethiopia is more than whether a factor like education explains a portion of the change in use behavior. For example, the authors note that secondary education explains about 1% of the observed change in LAPM use. What does that mean for policy efforts to increase the use of LAPMs? Does it mean a focus on secondary education? But then again, though significant, a 1% change, appears to be practically small and will have little practical value for any kind of policy efforts. I am hoping that the authors can engage these kinds of thoughts for their discussion section. | We appreciate your helpful comments. We addressed these issues in the revised manuscript. |
