# Supplementary material for: Determinants of change in long-acting or permanent contraceptives use in Ethiopia; A multivariate decomposition analysis of data from the Ethiopian demographic and health survey
Source: PLoS One. 2020 Jan 14;15(1):e0227218. doi: 10.1371/journal.pone.0227218 (PMC6959602; doi:10.1371/journal.pone.0227218)
Supplement: Attachment — Submitted filename: Reviewer_comment_R1.docx [file pone.0227218.s003.docx]

**Reviewer’s report**

**Title:** Determinants of change in long-acting and permanent contraceptive methods use in Ethiopia; a multivariate decomposition analysis of data from the Ethiopian demographic and health survey

**Version R1: Date**: 20^th^ November 2019

**Reviewer:** Samuel Bosomprah

**Reviewer’s report**

The paper sought to explain the change in LAPMs use over time. I believe that this is an important topic especially in developing countries where there is concern for population growth and its negative consequences on available resources and economic growth. The choice of decomposition analysis technique is appropriate to answer to the research question “why has LAMPs use change over time?”. The paper can be improved if the authors addressed the following concerns.

**Authors have addressed all my comments.**

**Discretionary Revisions:**

**Level of interest:** An article of importance in its field

**Quality of written English:** Standard

**Statistical review:** No, I am a statistician and have reviewed the statistical methods used.

**Declaration of competing interests:** I declare that I have no competing interest
