# Supplementary material for: Determinants of change in long-acting or permanent contraceptives use in Ethiopia; A multivariate decomposition analysis of data from the Ethiopian demographic and health survey
Source: PLoS One. 2020 Jan 14;15(1):e0227218. doi: 10.1371/journal.pone.0227218 (PMC6959602; doi:10.1371/journal.pone.0227218)
Supplement: Attachment — Submitted filename: Response to reviewers_2.docx [file pone.0227218.s004.docx]

**Response to Reviewers**

| **S.No** | **Editor’s/Reviewers’ comments** | **Authors’ response** |
| --- | --- | --- |
|  | **Reviewer 1** | |
| 1 | … The paper can be improved if the authors addressed the following concerns. | Thank you very much for your unreserved effort to improve our manuscript. But we cannot find the comments in the attachment or in the email. |
| **Reviewer 2** | | |
| **Introduction** | | |
| 1 | I am not sure I agree with the framing in the first sentence. Are unintended pregnancies an actual public health issue? Certainly, they are a social issue and perhaps an economic one. But a public health one I am not sure about that. Perhaps there are serious public health effects that arise out of a high rate of unintended pregnancies, but I am not sure characterizing the pregnancies themselves as a health issue is wise. | Thank you for the comment. In the text, we stated that unintended pregnancy is a global problem due to the consequences (abortion, maternal death, low maternal health service use, child mortality, maternal depression …) that follow it. As you stated it has also social and economic challenges to the woman, her family and the community. We revised the sentences so that it will reflect these issues (line 57 – 58 in the revised manuscript). |
| 2 | What is the national target for LAPMs (Line 80)? I think it would be a good idea to state this national target if it is known or cite the policy statement that makes it a national target. | Thank you for the comment. It is accepted and a statement describing this is included in the introduction section (line 77 - 78).  The national target of contraceptive prevalence rate at 2020 is 55%. Long acting or permanent methods are expected to be 50% of the method mix. That is about 22.5% women are expected to use one of the LAPM by 2020. |
| 3 | The introduction is missing a statement of aims and objectives. Traditionally, introductions will conclude with a paragraph illustrating what is intended for the rest of the manuscript. That appears to be missing in this revision. The authors get at that a bit with the last line of the paragraph (Line 84). | Thank you for the constructive comment. We now revised the last line so that it clearly shows the objective of the study (line 85 – 87 in the revised manuscript). |
| **Methods** | | |
| 1 | Personally, I believe the authors could do away with paragraph 1 in the methods or at least condense it a bit. The survey techniques employed for DHS are widely known. I think the authors could get by, by calling attention to the final reports which do a more detailed description of survey methods. | Thank you for the comment. We accept the suggestion and deleted some of the statements in the first paragraph. But, we do not believe that many people (who may read this paper) know the details of the DHS. We learned that while attending DHS fellowship program and train faculty staff on DHS data analysis. (Line 95 – 97 in the revised manuscript). |
| 2 | Line 100 - 106 Authors should consider providing a citation in support of the point of social desirability bias. Also, what makes the authors confident that married women or women in unions are not subject to social desirability biases when considering the issue of contraceptive use? There is an argument to be made for limiting the analysis to married/ women cohabiting with a partner, it is just not clear, at least in the text of the manuscript. The responses that the authors provided to my comment from the first review, when I raised a similar issue, could serve as a means of bolstering the argument here. | Thank you for the constructive comment. We are not concluding that married women are free from social desirability bias regarding long acting or permanent contraceptive methods use. But compared to unmarried women, it is definitely lower. Why social desirability bias on contraceptive studies may be higher among unmarried women is higher is related to the bias related to the bias reporting sexual activities. In Ethiopia, reporting sexual activity is taboo. When it is for unmarried women, it will be more taboo. In addition, the analysis is long acting or permanent contraceptive method use. Unmarried women are less likely to use these methods. To make the justification more clearly, we revised the statement. (Line 105– 108 in the revised manuscript). |
| 3 | The figure legend should be placed underneath the figure. Line 107 - 109 | Thank you for the comment. The guide to authors of the journal recommends writing figure captions this way. Kindly check the guide at <https://journals.plos.org/plosone/s/figures#loc-captions> |
| 4 | I can appreciate the authors wanting to describe the analytical methods the chose to use. But a step-by-step descriptor of STATA commands and the way they are applied is not necessary, at least in the context of this paper. For example, where the authors write, “First the 2005 EDHS data was opened.” And then later write, “Then, the 2016 EDHS was opened.” All of that is unnecessary in my view. All those lines can be condensed to something like, “2016 EDHS data was appended to 2005 data, using the ‘append’ command”. If the authors insist on providing detail about their STATA coding choices, then perhaps consider submitting the annotated STATA do file as a supplement to be included in an appendix. | Thank you for the comment. The comment is well accepted and revised now. (Line 149 in the revised manuscript). |
| **Result** | | |
| 1 | Line 166 - 167, “… and did not attend formal education.” This part of the sentence is confusing. Do the authors mean that the sampled women had no formal education or that they were not enrolled in school or some other kind of formal education at the time of the survey? I think it is the former. | Thank you for the comment. As you said, it is to say no formal education. It is now revised. (line 165 in the revised manuscript). |
| 2 | In Table 01, the authors present results from the chi-square analysis. However, they make no mention of the intention to do this type of analysis in the methods section of their manuscript. Chi-square is a good choice in this case, but the authors need to explain its use in the methods. Additionally, as a means of simplifying the table, why don’t the authors just summarize the chi-square findings in a manner similar to Table 03 (i.e., show the statistically significant findings using asterisk). | Thank you for the comment. At the end of the methods section, a sentences describing that we conducted chi-square test and its purpose is included in the revised version now.  (Line 155 – 157 in the revised manuscript). The significance values of the chi-square tests in table 1 are also modified in the revised version.  (Line 169 – 170 in the revised manuscript). |
| 3 | I recommend the authors take a look at Table 02. From the table, it is clear that more women are using LAPMs in 2016 than were using in 2005. However, when breaking this down by age categories the change is not as large as you do see in other categories. For 'age' we see a percent change from 2005 to 2016 of about 1.0 while for all the other variables (i.e., residence, religion, etc.) we are seeing percentage changes by categories that are very high. Why does the break down by age not account for these fairly large changes, is it an error? | Thank you very much. It was a very important view. We learned that there was error on the figures related to the 2016. We recalculated the figures and revised the table.  (table 2) |
| 4 | Also, for Table 2 it would be a good idea to have a row at the bottom that accounted for all the women in the sample. That is the information provided in Lines 184 - 185 in the section on decomposition. | Thank you for the constructive comment. The sample size is now included with the year in brackets. (table 2) |
| **Discussion** | | |
| 1 | Line 226 - 227 The sentence beginning with, “About 10% of the increase…”, is confusing. | Thank you for the clarification. As stated, the change is due to the change in the effect of different characteristics of women. Overall, about 44% of the change is due to the effect of age on LAPM uptake. When this change is further decomposed, about 10% from this total is due to the effect of women aged 35 to 49. |
| 2 | Line 228 - 229 I am not convinced by this argument, not that women in the 35-49 age bracket may want to have fewer children. But that the authors don’t put forth an explanation. Why would women in that age bracket seek fewer children on average? | Thank you for the clarification. Women in this age group may have reached their desired level of fertility. Therefore, they may not want more children. A phrase is added to make the sentences more clear. (line 228 – 229 in the revised manuscript). |
| 3 | Line 232 - 242 I think citing some sources may help to strengthen the authors’ arguments here. I assume that there are documented studies or interventions showing that women with an education are more likely to use LAPMs or modern contraceptives in general. For example, in line 238 where the authors suggest that LAPMs may help make Ethiopian women more economically productive, I am confident that there are economic studies demonstrating that women with a better grasp on their fertility, are more economically productive. Citing something like that gives the point the authors make in this paragraph more credibility and moves their arguments from the area of speculation. | Thank you for the comment. References are added now.  (line 235 – 236 in the revised manuscript). |
| 4 | Line 243 - 256 Similar to my comment above, I am also confident that there are interventions and studies detailed in the literature which demonstrate that some kind of home visit by some kind of medical provider/ health worker, can serve to alter behavior towards LAPMs. Citing such studies in support of the arguments here strengthens the paper in my view. | Thank you for the comment. Findings related to the effectiveness of home visit are included. Literatures that show home based counseling is better than health facility is also included. (Line 250 in the revised manuscript). |
| 5 | Line 253 - 256 I appreciate that the authors making this inclusion into the paper. I would suggest that the authors resist using such definitive language. Say, “The findings from this study may inform” as opposed to, “The findings from this study will inform.” There is no guarantee that policymakers will take up your work. | Thank you for the comment. It is revised now.  (Line 252 in the revised manuscript). |
| 6 | Did the authors remove their description of study limitations? | Thank you for the comment. The limitation is still there. |
| 7 | Are there interventions that could benefit from the results of your analysis? If there are some on going on some planned, it may help to mention them in the discussion. Or the authors could suggest an intervention based on their findings, as something that merits further research. | Thank you for the comment. A recommendation of these interventions is included at the end of the conclusion.  (line 263 – 265 in the revised manuscript). |
| 8 | Another potential issue of discussion is the rate of LAPMs among married men/ or men cohabiting with women. It is not the focus of your paper. However, if the argument is about informing policymaking and filling in gaps in our understanding, then it may be useful to bring up men’s use of LAPMs particularly vasectomy. The authors could bring it up in the context of future studies that need to be done in the area. | Thank you for the comment. Vasectomy is very low in Ethiopia. But if there were women who had undergone vasectomy, his wife was considered as using permanent methods. The data in DHS considered this. Therefore, vasectomy is included in the permanent methods. |
